# Assessment of caregiver expectations of physician communication in a pediatric setting

**DOI:** 10.1186/s12913-020-05262-x

**Published:** 2020-05-11

**Authors:** Tyler Lee, Julie Cui, Hinette Rosario, Didja Hilmara, Kate Samuelson, Emery C. Lin, Victoria A. Miller, Henry C. Lin

**Affiliations:** 1 Division of Gastroenterology, Hepatology, and Nutrition, Children’s Hospital of Philadelphia, Philadelphia, PA USA; 2 Department of Medicine, College of Medicine Drexel University, Philadelphia, PA USA; 3 Division of Adolescent Medicine, Children’s Hospital of Philadelphia, Philadelphia, PA USA; 4 Perelman School of Medicine, University of Pennsylvania, Philadelphia, PA USA

**Keywords:** Physician communication, Pediatrics, Patient and caregiver satisfaction

## Abstract

**Background:**

In pediatrics, communication often occurs through an intermediary such as a caregiver. The goal of this study is to assess caregiver communication expectations and determine if meeting expectations influences caregiver satisfaction or instruction retention.

**Methods:**

A survey study was performed at the Children’s Hospital of Philadelphia. Before the visit, caregivers completed a survey on communication expectations, Caregiver Expected Kalamazoo Essential Elements Communication Checklist (Caregiver Expected KEECC). After the visit, caregivers were surveyed on their perception of physician communication (Caregiver Perceived KEECC) and satisfaction. Caregivers were contacted 1 week after the clinic visit to assess instruction retention. Meeting of caregiver expectation was calculated by the difference between Caregiver Expected and Caregiver Perceived KEECC scores.

**Results:**

112 caregivers participated in the study. There was no significant difference in Caregiver Expected KEECC versus Caregiver Perceived KEECC score (4.39 vs 4.56). Caregiver communication expectations were exceeded in 51.5% of the visits. Communication expectations were exceeded more among caregivers with at a college education (*p* <  0.01) and more among White caregivers (p <  0.01). The average caregiver satisfaction score with the clinic visit was 4.67. Higher satisfaction scores were observed in caregivers who had their communication expectations met or exceeded (p <  0.01). Caregivers with communication expectations exceeded had higher percentage recall of physician instructions (p <  0.01).

**Conclusions:**

Caregiver communication expectations may be influenced by demographic factors. Communication expectation affects visit outcomes including caregiver satisfaction and instruction retention. Therefore, physicians need to be cognizant of caregiver communication expectations, which can impact quality of the healthcare experience.

## Background

Clear and effective communication between a physician and patient is a vital aspect of medicine. Effective communication allows for directed discussion of health issues to inform diagnoses and treatment plans, and establishes positive and healthy relationships with patients [1, 2]. In pediatrics, patient-physician communication often occurs through a third party (parent or caregiver) instead of directly with the patient. In this communication model, the caregiver serves as a patient advocate, and physicians rely on the caregivers to describe the patient’s problems. Relying on caregivers as historians presents a challenge as the caregiver may have trouble accurately describing the patient’s medical symptoms.

Communication in healthcare is a complex process and many factors may influence communication between the physician and patient including the structure, goal, and outcome of the clinical interaction. Structure of the clinical interaction is dependent on physician behaviors, patient behaviors, and situational factors (such as prior established relationship or physician workload) at the time of the clinical interaction [3]. Patient behavior can influence the quality of communication as anxiety and fear may make patients feel less comfortable to share the information necessary for the physician to make a proper diagnosis [4]. Social factors or health beliefs may lend to patient hesitation of sharing necessary information in hopes of asserting their own perspectives. Physician-based factors such as avoidance behaviors, nondisclosure of information, or discouragement of collaboration may also influence the quality of communication [2, 3]. For example, thoughts of litigation, patient verbal abuse, and unrealistic patient expectations can lead to physician avoidance behaviors of discussing non-health related topics that may be important to the treatment of patients [5]. Dedicated training in interview skills [6] and an awareness of patient communication behaviors and expectations can help physicians develop more effective communication skills.

In pediatrics, the physician-caregiver-patient relationship is often dynamic, as the physician must determine when to communicate with the parent or the patient or both. Children can be quite perceptive and some observational studies suggest that children are not only interested in their own clinical information but may actually remember information better than adults [7, 8]. Traditionally, there are three physician communication dimensions to the physician–caregiver–child dynamic: informativeness, interpersonal sensitivity, and partnership building. Informativeness is the quality of medical information provided to the caregiver and child. Interpersonal sensitivity relates to physician behaviors that reflects interest in the caregiver’s and patient’s feelings and concerns. Partnership building is the manner in which the physician involves the caregiver and patient to participate in the interaction and share their perspectives [9, 10]. Central to these dimensions of physician communication is the ability to clearly express thoughts and explanations in a manner that can be understood. To avoid any confusion or misunderstandings, physicians must possess firm control over the diction and syntax of their communication. Ultimately, the manner a physician informs a caregiver about their child’s health, may not only affect caregiver comprehension, but may also impact further relations with the caregiver and patient [11].

Effective physician interpersonal and communication skills can engage both the caregiver and patient in medical decision making which can lead to positive clinical outcomes [12] and may also and help avoid dissatisfaction that results from communication breakdown in the physician-patient relationship [2]. Generally, a patient-centered encounter encourages communication and leads to higher patient satisfaction [2, 5, 13–15]; and in pediatrics, this has led the studies on understanding the child’s perspective and engaging the child to become a more active participant during the clinical visit [8, 16–18]. However, as much of pediatric communication revolves around the caregiver, many studies of satisfaction in pediatrics focuses on the caregiver. Parental satisfaction with their child’s healthcare has been shown to be correlated with parental perceptions of the physician’s informativeness and interpersonal sensitivity [9]. Nonetheless, communication preferences vary by person, and preferences of physician communication style can depend on individual characteristics such as anxiety or education level [19, 20]. Therefore, understanding caregiver communication expectations or preferences could potentially improve caregiver satisfaction. For example, Levine et al. (2019) addressed the communication needs of the patient and caregiver in a pediatric oncology setting, identifying a parental desire for increased focus on discussion of diagnosis on family life and long-term quality of life [21].

Various assessment tools have been developed to quantify effective communication in healthcare. Howells et al. (2010) mapped physician communication performance to two domains: clinical skills and communication behavior [22]. The tenets of effective, patient-centered communication behavior include exchange of information, support of patient self-management and decision-making, enhancement of physician-patient relationship, and management of uncertainty and emotions [23, 24]. However, the challenge is to identify what the individual patient deems as quality communication behavior during a specific clinical interaction [24]. Understanding the expectations and preferences of patients and caregivers can help physicians learn how to adapt their communication strategies to individual communication needs.

The goals of this study are to: (1) assess caregivers’ communication expectations in a pediatric outpatient clinical visit, (2) determine if caregiver demographics play a role in whether physicians meet caregiver communication expectations, and (3) determine if meeting caregiver communication expectations impacts caregiver instruction retention or satisfaction.

## Methods

A survey study was performed at the Children’s Hospital of Philadelphia, Pediatric Gastroenterology outpatient clinic from February 2016 to August 2016. The patient demographic at the pediatric gastroenterology clinic ranges from infancy to 17 years old and includes mostly families from the greater Philadelphia area. Per the most recent American Community Survey, the racial composition of Philadelphia is 42% Black or African American, 42% White, and 7% Asian.

This survey study was designed to consist of one pre-visit survey, two immediate post-visit surveys, and one phone survey administered one week after the clinic visit. The participants of this study were the caregivers of patients seen in the pediatric gastroenterology clinic. Inclusion criteria for the study included English as a primary language, the patient being between the ages of 0 to 17 years old, and caregivers who were able to meet the follow-up requirements. Exclusion criteria consisted of non-English speaking or patients older than 18 years of age. In this pilot study on caregiver perception of communication, the study population was a sample of convenience and sample size was not calculated. Previous published studies on communication behavior have ranged in size from 20 up to 458 [8, 16, 22, 25, 26] to determine an estimated target sample size.

Caregivers were approached in the waiting room prior to their child’s visit and consented for participation in the study. Once consented, caregivers were administered a pre-visit survey. The pre-visit survey consisted of caregiver and patient demographic information as well as caregiver communication expectations of the physician (Caregiver Expected KEECC Survey). At the conclusion of the visit, caregivers were administered two additional surveys. One survey assessed the caregiver’s perception of the physician’s communication (Caregiver Perceived KEECC Survey). The other survey assessed the caregiver’s satisfaction with the clinic visit (Communication PSQ-18). All surveys were completed by hand and then transcribed to RedCap by a study coordinator after the visit.

The standard practice of the gastroenterology clinic is to provide caregivers with written patient instructions at the end of each visit that summarizes what was discussed and the next management steps. These instructions are typically verbally reviewed by the physician at the close of the visit. To assess caregiver instruction retention, these instructions were recorded in RedCap. A follow-up phone call to the caregiver was made 7 days after the clinic visit. Two additional follow-up phone call attempts were made if the caregiver was not reached. A phone survey was administered to the caregivers, which consisted of asking caregivers if they received written instruction at the end of the visit and if they could spontaneously recall the written physician instructions provided at the close of the clinic visit. Instruction retention was reported as a percentage and based on whether or not caregivers could recall the physician instructions provided on the after visit summary. Caregiver responses were recorded on RedCap.

### Survey instruments

The Kalamazoo Essential Elements Communication Checklist (KEECC) is a validated as a measure of communication skills in the medical setting [27]. The KEECC consists of 24 items corresponding to one of the seven essential elements of physician communication: Building a Relationship (BR), Opening the Discussion (OD), Gathering Information (GI), Understanding Patient Perspective (UPP), Sharing Information (SI), Reaching an Agreement (RA), and Providing Closure (PC). Each item is scored on a Likert scale from 1 to 5. To assess caregiver expectations of physician communication in the pre-visit survey (Caregiver Expected KEECC Survey), each of the 24 items of the KEECC was rephrased following a standard format. For example, the item “greets and shows interest in the patient’s family” was rephrased as “You expect that your child’s physician to greet and show interest in the patient’s family?”. Likewise, to assess caregiver perception of physician communication in the post-visit survey (Caregiver Perceived KEECC Survey), each item was rephrased in the following format: “During today’s visit, your child’s physician greeted and showed interest in the patient’s family”. The KEECC Cronbach α value was 0.98 for the Caregiver Expected KEECC Survey. The Caregiver Perceived KEECC Survey had a Cronbach α value of 0.98.

Caregivers’ communication satisfaction was measured using a previously validated version of the Patient Satisfaction Questionnaire 18 (Communication PSQ-18) that was tailored specifically to assess communication satisfaction [28, 29]. Cronbach α value for the Communication PSQ-18 was 0.96.

Items throughout the Caregiver Expected KEECC, Caregiver Perceived KEECC, and Communication PSQ-18 surveys were set on a 5 point Likert scale with a score of 5 indicated the most positive meaning such as “Excellent” or “Strongly Agree”, and a score of 1 indicating the most negative meaning such as “Poor” or “Strongly Disagree.” An average overall score out of 5 was calculated for each survey. In addition, for the Caregiver Expected KEECC and the Caregiver Perceived KEECC, average subcategory scores were calculated for each of the 7 essential elements subcategories of the survey.

Descriptive statistics were used to summarize findings presented as mean and standard deviation. For quantitative analysis, t-tests were run to see if there was a significant difference in the Caregiver Expected KEECC between different variables: race (white vs non-white), education (college vs no college), and gender (male vs female). The same analysis was also performed for the Caregiver Perceived KEECC score and the Communication PSQ-18. Paired t-tests were run between the Caregiver Expected KEECC and the Caregiver Perceived KEECC to see if there was any difference between expected and perceived physician communication scores.

To assess if caregiver communication expectations were met, the difference between the Caregiver Expected KEECC and Caregiver Perceived KEECC scores was calculated with a delta of zero indicating that communication expectations were met. A positive delta indicated “exceeding of communication expectations” and a negative delta indicated “not meeting of communication expectations”. Subgroup analyses were run to see if there were significant differences in the outcome variables (Communication PSQ-18 score or instruction recall percentage) between caregivers with expectations met or net met among different subgroups: (white vs non-white), education (college vs no college), and gender (male vs female).

Ethics approval was obtained for this study via review by the Children’s Hospital of Philadelphia’s Institutional Review Board (IRB). In consultation with the IRB, to protect patient privacy and confidentiality, the video recordings were stored on a secure, password protected server with the initial recording destroyed. All information was de-identified with any identifiers stored in separate documents on a separate password-protected server and participants were informed that they had the right to terminate participation in the study at any time.

## Results

### Demographics

A total of 171 caregivers were approached and 112 participants consented to participate in the study, 97 of whom completed all the surveys for a response rate of 86.6%. Of the 97 visits, 47 were new patient visits and 50 were follow-up visits. Patients ranged in age from infancy to 17 years old, with 25% infants or toddlers, 14.3% preschoolers (3–4.9 years), 34.8% grade-schoolers (5–12 years), and 25% teenagers (13–17 years). A total of 11 different attending physicians with between 1 to 15 years of experience saw patients during the clinic visits. There were 8 female and 3 male physicians.

Caregiver demographics were optional on the survey. Of those reported, 60 caregivers were White (61.8%) and 35 were non-White (36.1%). 82.5% of caregivers were female and 17.5% were male. The majority of caregivers (69.1%) reported an education level of at least completing college, while 26.8% did not have a college degree (Table 1).

**Table 1:** Caregiver Demographics (*n* = 97)

	n*	%
**Education Level**		
College Degree	67	69.1%
No College Degree	26	26.8%
**Race**		
White	60	61.8%
non-White	35	36.1%
**Gender**		
Female	80	82.5%
Male	17	17.5%

*Caregiver demographics were option on the survey, and not all caregivers provided a response in this section of the survey

### Caregiver communication expectations

Caregiver expectations of physician communication were assessed using the Caregiver Expected KEECC survey that was administered prior to the clinic visit. The average overall Caregiver Expected KEECC score was 4.39 out of 5 (σ = 0.84). Of the 7 essential elements of communication subcategories of the Caregiver Expected KEECC, the lowest average subcategory score was of Understanding Patient Perspective, 4.18 (σ = 0.97) (Table 2). Caregiver expectations for Understanding Patient Perspective was significantly lower compared to average scores of 3 other communication subcategories: Building a Relationship (4.52, *p* = 0.01), Sharing Information (4.58, *p* <  0.01), and Providing Closure (4.49, *p* = 0.02).

**Table 2:** Average Scores of KEECC Categories (n = 97*)

KEECC Categories	Caregiver Expectation KEECC Score	Caregiver Perceived KEECC Score
Building a Relationship	4.52 ± 0.94	4.48 ± 0.81
Opening a Discussion	4.32 ± 0.85	4.54 ± 0.74
Gathering Information	4.32 ± 0.97	4.53 ± 0.86
Understanding Patient Perspective	4.18 ± 0.97	4.58 ± 0.69
Sharing Information	4.58 ± 0.85	4.60 ± 0.79
Reaching an Agreement	4.34 ± 0.90	4.56 ± 0.89
Providing Closure	4.49 ± 0.84	4.62 ± 0.77
**Overall Average KEECC Score**	**4.39 ± 0.84**	**4.56 ± 0.70**

*Participants who completed both Preferred KEECC and Perceived KEECC surveys

The average Caregiver Expected KEECC score reflects caregiver expectations of physician communication. Caregivers who did not complete college had significantly higher communication expectation than caregivers who completed at least a college education (4.78 vs 4.39, t(89) = 3.87, p <  0.01). Non-white caregivers had significantly higher general communication expectations compared to White caregivers (4.76 vs 4.36, t(92) = 3.93, p <  0.01). The Caregiver Expected KEECC score did not vary by caregiver gender (Table 3). There was no significant difference in the Caregiver Expected KEECC scores between caregivers at a new patient versus a follow-up clinic visit.

**Table 3:** **Survey Scores by Caregiver Demographics (*n*** **= 97)**

	Education			Gender			Race			Visit Type		
	College	No College	***p***-value	Male	Female	p-value	White	non-White	p-value	New	Follow-up	p-value
**Caregiver Expected KEECC**	4.39 ± 0.66	4.78 ± 0.30	< 0.01	4.20 ± 0.99	4.57 ± 0.47	0.15	4.36 ± 0.67	4.76 ± 0.33	< 0.01	4.42 ± 0.71	4.59 ± 0.65	0.18
**Caregiver Perceived KEECC**	4.56 ± 0.72	4.57 ± 0.60	0.97	4.54 ± 0.52	4.58 ± 0.71	0.8	4.66 ± 0.51	4.41 ± 0.89	0.12	4.53 ± 0.72	4.61 ± 0.46	0.57
* Delta	0.17	−0.21	0.02	0.33	0.01	0.24	0.31	−0.36	< 0.01	0.11	0.02	0.61
**Communication Satisfaction**	4.70 ± 0.62	4.58 ± 0.65	0.43	4.70 ± 0.58	4.67 ± 0.64	0.87	4.80 ± 0.44	4.47 ± 0.82	0.03	4.65 ± 0.63	4.70 ± 0.62	0.69

* Delta is calculated by the following formula: Caregiver Perceived KEECC score – Caregiver Expected KEECC score

### Caregiver communication perception

Caregiver perception of physician communication during the clinic visit was assessed using the Caregiver Perceived KEECC survey which was administered after the visit. The average overall score for Caregiver Perceived KEECC was 4.56 (σ = 0.70). Of the 7 Caregiver Perceived KEECC subcategories of communication, caregivers rated Building a Relationship as the lowest perceived physician communication characteristic during the visit, 4.48 (σ = 0.81) (Table 2). However, this score was not significantly different from the other 6 KEECC subcategory scores. Caregiver Perceived KEECC mean scores did not vary by caregiver education level, race, gender, or visit type (Table 3).

### Meeting of caregiver communication expectations

There was no significant difference in mean Caregiver Expected KEECC score compared to mean Caregiver Perceived KEECC score. To assess whether or not caregiver expectations were met, the delta between the Caregiver Expected KEECC and Caregiver Perceived KEECC scores was calculated. Caregiver communication expectations of the visit were exceeded in 50 of the visits (51.5%) as defined by a positive difference or delta between the Caregiver Perceived KEECC and the Caregiver Expected KEECC scores. The range of positive delta was 0.01 to 2.95 with an average positive delta of + 0.58 (σ = 0.58). Twenty-one caregivers had their communication expectations just met (delta = 0). Communication expectations were not met for 26 caregivers during the visit (26.8%), with the negative delta ranging from − 0.01 to − 2.79 (μ = − 0.84, σ = 0.67).

Caregiver education level and race were found to be significant predictors of meeting of caregiver communication expectations. With an average positive delta of + 0.17, caregivers with at least a college education had their communication expectations exceeded significantly more than caregivers without a college education (average delta of − 0.21), t(73) = 2.42, *p* = 0.02. Only 9 out of 26 caregivers who did not complete a college education had a positive delta. The average Caregiver Expected KEECC score among caregivers who did not complete a college education was 4.78 as compared to their average Caregiver Perceived KEECC score of 4.58. While this difference was not statistically significant, the average negative delta of − 0.21 suggests that these caregivers’ communication expectations may not have been met.

White caregivers had their communication expectations exceeded significantly more than non-White caregivers (+ 0.31 versus − 0.35, t(70) = 3.99, *p* <  0.01). Only 10 out of 35 non-White caregivers had a positive delta. With an average negative delta of − 0.35 among non-White caregivers, this finding suggests that their communication expectations may not have been met. The average Caregiver Expected KEECC score among non-White was 4.76, which is statistically significantly different as compared to White caregivers (4.36, *p* <  0.01). However, the average Caregiver Perceived KEECC score did not vary between White and non-White caregivers.

Meeting of caregiver communication expectations did not vary by caregiver center or visit type. Both new patient visits (+ 0.11) and follow-up visits (+ 0.02) had an overall positive average delta.

### Caregiver outcomes

The impact of physician communication on caregiver satisfaction and caregiver retention of clinic visit instructions was analyzed. The average Communication PSQ-18 score was 4.67 (σ = 0.62) (Table 4). Significantly higher communication specific PSQ scores were observed in caregivers who had their communication expectations met as compared to caregivers whose communication expectations were not met (4.92 vs 4.17, t(35) = 5.14, p <  0.01).

**Table 4:** Average Scores of Communication Specific PSQ-18 Statements (n = 97)

Communication Specific PSQ-18 Statements	Average Score ± STD
Greeting you warmly; calling you by the name you prefer; being friendly; never crabby or rude	4.66 ± 0.68
Treating you like you’re on the same level; never ‘talking down’ to you or treating you like a child	4.64 ± 0.84
Letting you tell your story; listening carefully; asking thoughtful questions; not interrupting you while you’re talking	4.66 ± 0.74
Showing interest in you as a person; not acting bored or ignoring what you have to say	4.68 ± 0.65
Encouraging you to ask questions; answering them clearly; never avoiding your questions or lecturing you	4.69 ± 0.65
Using words you can understand when explaining your problems and treatment; explaining any technical medical terms in plain language	4.70 ± 0.63
**Overall Average Score**	4.67 ± 0.62

Caregiver instruction retention was determined by a follow-up phone call to the caregiver one week after the clinic visit. 67 out of the 97 caregivers (69.1%) were successfully contacted. Caregivers whose communication expectations were exceeded had significantly higher instruction recall percentage of physician instructions compared to caregivers whose communication expectations were just met (85% vs 59%, t(33) = 1.69, *p* < 0.01) and not met (85% vs 56%, t(13) = 1.77, *p* = 0.04). Caregiver recall percentage did not vary by education level, gender, race, or visit type (Table 5).

**Table 5:** Caregiver Outcomes by Selected Demographics*

Caregiver Demographic	Communication Specific PSQ-18 Score	Instruction Recall
	(Average ± STD)	(Average % ± STD)
**No College Degree**		
Expectation Met/Exceeded	4.95 ± 0.11	72% ± 0.030
Expectation Not Met	4.07 ± 0.73	58% ± 0.39
p-value	< 0.01	0.41
**College Degree**		
Expectation Met/Exceeded	4.86 ± 0.32	75% ± 0.32
Expectation Not Met	3.97 ± 0.96	58% ± 0.32
p-value	< 0.01	0.24
**White**		
Expectation Met/Exceeded	4.91 ± 0.26	78% ± 0.28
Expectation Not Met	4.30 ± 0.64	60% ± 0.39
p-value	< 0.01	0.31
**non-White**		
Expectation Met/Exceeded	4.91 ± 0.23	62% ± 0.42
Expectation Not Met	4.02 ± 0.66	53% ± 0.38
p-value	< 0.01	0.63

* When looking at the cohort as a whole (n = 97), caregivers whose communication expectations were exceeded had significantly higher recall percentage of physician instructions compared to caregivers whose communication expectations were just met (85% vs 59%, p < 0.01) and not met (85% vs 56%, p = 0.04)

The caregiver Communication PSQ-18, Caregiver Expected KEECC, and Caregiver Perceived KEECC scores did not vary by physician experience (< 5 years versus >/=5 years) or by physician gender (male versus female) as there was no significant difference in these scores when stratified by these groups (Table 6). Likewise, there was no significant difference in Communication PSQ-18, Caregiver Expected KEECC, and Caregiver Perceived KEECC scores among caregivers of different patient age groups (infant, toddler, school age, and teenager).

**Table 6:** Caregiver Survey Scored by Selected Patient and Physician Demographics*

	Caregiver Expected KEECC	Caregiver Perceived KEECC	Caregiver Satisfaction
**Patient Age**			
**0–2 years**	4.27 ± 0.92	4.36 ± 0.89	4.55 ± 0.61
**3–4 years**	4.30 ± 1.00	4.76 ± 0.37	4.86 ± 0.29
**5–12 years**	4.50 ± 0.68	4.50 ± 0.77	4.60 ± 0.79
**13–17 years**	4.38 ± 0.95	4.73 ± 0.40	4.79 ± 0.40
**Physician**			
**Female**	4.41 ± 0.82	4.54 ± 0.75	4.66 ± 0.67
**Male**	4.34 ± 0.90	4.62 ± 0.54	4.70 ± 0.49
**< 5 years experience**	4.57 ± 0.50	4.67 ± 0.56	4.77 ± 0.51
**>/=5 years experience**	4.12 ± 1.13	4.38 ± 0.85	4.52 ± 0.74

*Results presented as mean ± standard deviation

## Discussion

This study assessed caregiver communication expectations and caregiver perception of the quality of physician communication during the clinic visit. The same assessment tool (KEECC) was used to assess caregiver expectations and caregiver perception of communication, which allowed for comparison of the Caregiver Expected KEECC and Caregiver Perceived KEECC scores to determine if caregiver communication expectations were met. Understanding caregiver communication expectations and meeting of caregiver communication needs can facilitate effective communication.

Patient-centered communication is one way to improve effective communication and depends on a physician’s ability to empathize with their patients. Displaying empathy during patient encounters allows the physician to gain insight on a patient’s perspectives and facilitates the patient’s perception as an active participant in their healthcare [30]. This approach helps build a positive relationship, creating patient confidence in the physician and helps the patient feel comfortable to disclose the information needed for medical management [2]. In pediatrics, a part of this patient-centered approach should include understanding caregiver communication expectations. Possible strategies to query for caregiver communication expectations include pre-visit screening questions on the caregiver’s perception of the role of the physician or incorporating these questions into a routine part of the visit. Acknowledging that individuals have varied communication needs and signaling a desire to understand how best to ascertain necessary medical information from the caregiver to help is key.

Based on KEECC subcategory analysis, Sharing Information was the most valued communication subcategory by caregivers, which could be explained by the fact that caregivers are providing necessary information and concerns to seek medical attention for their child, which is usually the main reason for the clinic visit. Caregivers may have more control over the communication process when sharing information, as caregiver input drives physician responses and management. In some instances, a caregiver led discussion may save time during clinic visits and provide the necessary details for a physician to make a proper diagnosis [31]. Other high scoring KEECC subcategories were Building a Relationship and Providing Closure, which could be valued communication characteristics of a visit because these subcategories demarcate the beginning and end of a visit respectively. Building a Relationship could be a surrogate for a first impression of the physician and can dictate how the medical discussion progresses. Providing Closure summarizes the key points of the visit.

Within pediatrics, one study suggested 6 domains of physician communication that both children and parents identified to be influential in their quality of care: relationship building, demonstration of effort and competence, information exchange, availability, appropriate level of child and parent involvement, and coordination of care [16]. While these communication domains were identified based on the perception of physician communication received, many similarities are seen when compared to the caregiver reported communication expectations in our study. Caregiver emphasis on building a relationship, sharing information, and providing closure suggests that the communication strategies employed by physicians may match caregiver communication expectations.

Expected physician communication varies among individuals and the challenge facing physicians is meeting the communication expectations of their patients. In our study, caregiver demographics appeared to play a role in Caregiver Expected KEECC scores. Caregivers who did not complete a college degree reported significantly higher communication expectations, which could be due to concern about a perceived knowledge discrepancy or a lack of understanding of the medical terminology. Functional health literacy can allow for medical conversations to be less complicated and more straightforward [32]. However, physicians may overestimate patient or caregiver health literacy, which could lead to confusion and frustration [33]. It is understandable for caregivers to have an expectation that the physician communicates in a way that can be easily understood—at a comfortable health literacy level. Conceivably, caregivers without a college degree may feel more intimidated by or at risk of not understanding the physician. Therefore, to meet caregiver communication expectations, it is imperative for a physician to consider and find a way to assess caregiver health literacy.

Caregiver communication expectations also varied by race, with non-White caregivers having significantly higher communication expectations than White caregivers. The demographics of non-White caregivers in our study included 60% Black or African-American, 17.1% Hispanic, 14.3% Asian, and 8.6% Other. The reason for this difference in caregiver communication expectations is unclear, but it is possible that communication expectations by caregivers are influenced by past experiences or existing biases with the healthcare system. One study that compared patient-physician communication between White and Black patients reported that with Black patients, physicians were “more verbally dominant” and the visits were “less patient centered” than with White patients [26]. In our study, the caregiver perceived quality of physician communication as measured by the Caregiver Perceived KEECC score was the same irrespective of race.

Meeting of caregiver expectations can be defined as a function of expected communication and perceived communication. In our study, the final Caregiver Perceived KEECC score did not vary by caregiver demographics, suggesting that physicians in the study provided the same level of communication regardless of the caregiver’s education level, gender, or race. Therefore, meeting communication expectations primarily depended on the caregiver’s initial communication expectation, as measured by the Caregiver Expected KEECC. There were significant differences in Caregiver Expected KEECC scores when compared by caregiver education level (college degree vs no college degree) and by caregiver race (White vs non-White). The reason for this higher communication expectation among non-White caregivers or caregivers without a college degree is unclear, but physicians should be cognizant of potential existing biases that may impact physician-caregiver-patient interactions.

Physician and patient characteristics may also influence caregiver communication expectations and perceptions. Patient age or maturity may affect caregiver desire for the physician to engage the patient in the medical discussion. Physician characteristics such as level of experience or gender should also be considered. For example, there may be an expectation that an experienced physician is more knowledgeable and therefore able to communicate more effectively with the caregiver. One study on communication between physician and patient relatives reported that physician gender and discipline affected communication attitude to the patient [34], which can affect the overall healthcare experience. Being attuned to caregiver communication expectations can help the physician improve the quality of healthcare for the patient and caregiver.

In pediatrics, aside from clinical outcomes, surrogate markers of quality of care include caregiver satisfaction, caregiver adherence to physician instructions, and caregiver perception of quality communication. With our study, among caregivers who had their communication expectations exceeded, significantly higher communication specific PSQ scores and percentage recall of physician instructions were observed. Exceeding caregiver communication expectations may allow caregivers to associate a positive emotion with the visit (i.e., caregivers being impressed with how clearly physicians communicated a complex diagnosis). Positive emotions can help one to reflect on the overall experience, resulting in greater recall [35]. In this case, caregivers who associated positive emotions with their clinical visit experience had greater recall of instructions from their physician. Ultimately, effective communication can positively affect caregiver experience, and physicians should consider ways to assess meeting of caregiver communication expectations.

Limitations of this study are related to the survey nature of the study and include response bias as most of the data was obtained from participant self-reports [36–38]. It is possible that social desirability could lead to caregiver reporting of higher than actually perceived communication or satisfaction scores. Another limitation is that while the surveys used in this study have previously been validated, they were modified for the pediatric setting to be administered to caregivers; but the Cronbach α for each survey was 0.96 or greater. The assessment of instruction recall was limited by recall bias; and as this study included 11 different physicians, the various physician communication strategies may have affected caregiver instruction comprehension. Reporting bias should be considered as it is possible that participants were reading the list of instructions instead of providing a mental recall, which would affect the instruction recall metric. Physician communication variability may also influence the perception of communication quality scores as certain physicians may be perceived as more better communicators, but there was no variation in the KEECC or PSQ scores by physician. Ultimately though, meeting of caregiver expectations is dependent on caregiver interpretations of the quality of communication and not solely by the physician. In addition, physician performance bias must be considered, as physicians may have been aware of caregiver participation in the study, which could have influenced physician communication behavior and thus the Caregiver Perceived KEECC score. Additional limitations to consider include the lack of a sample size calculation, exclusion of non-English speaking caregivers, unclear reasons for potential participants to decline participation in the study, and individual variation in communication expectations.

In conclusion, our study suggests that communication expectation is a driving force in determining visit outcomes as defined by caregiver satisfaction and instruction retention. While certain caregiver demographics may predispose to different initial communication expectations, in general, physicians provide a standard quality of communication during the clinic visit that is perceived relatively the same by participants, regardless of demographics. However, as meeting of communication expectations can affect caregiver experience, physicians should be aware of these potential differences in communication expectations and consider strategies to assess meeting of caregiver communication needs.

Based on this pilot study, we are interested in studying the outcomes associated with effective caregiver communication. We are particularly interested in further studying this concept of meeting of caregiver expectations. As caregiver demographics of race and education level seemed to impact Caregiver Perceived KEECC score, we would like to further investigate this potential relationship by studying underlying perceptions of communication in the caregiver-physician dyad through focus groups and surveys. We plan to use this pilot data to determine sample size to properly power studies to further assess these relationships. In addition, we are interested in assessing the various factors that impact caregiver instruction recall through semi-structured interviews and surveys as well as to determine how communication affects instruction recall.

Additional studies are needed to further explore the relationship between meeting of caregiver communication expectations and impact on experience and outcome in the pediatric setting.

## Data Availability

The datasets used and/or analyzed during the current study are available from the corresponding author on reasonable request.

## Abbreviations

KEECC: Kalamazoo Essential Elements Communication Checklist
IRB: Institutional Review Board
Communication PSQ-18: Patient Satisfaction Questionnaire 18
BR: Building a Relationship
OD: Opening the Discussion
GI: Gathering Information
UPP: Understanding Patient Perspective
SI: Sharing Information
RA: Reaching an Agreement
PC: Providing Closure
